# Mixed lesion of traumatic pseudoaneurysm and pyogenic granuloma on a digit

**DOI:** 10.1080/23320885.2023.2228887

**Published:** 2023-06-29

**Authors:** Toshifumi Yamashiro, Yusuke Hachisu, Ryuichi Azuma

**Affiliations:** a Department of Plastic and Reconstructive Surgery, National Defense Medical College, Tokorozawa, Saitama, Japan; b Department of Plastic Surgery, Japan Self-Defense Forces Sapporo Hospital, Sapporo, Hokkaido, Japan

**Keywords:** False aneurysm, digital artery, hand surgery, microvascular surgery, ultrasound

## Abstract

Traumatic aneurysms occurring in the digit are extremely rare. We report a case of a traumatic pseudoaneurysm arising from a terminal branch of the finger artery and presenting as a mixed lesion with pyogenic granuloma that was exposed to the outside of the body and treated surgically.

## Introduction

Traumatic aneurysms of the upper extremities generally occur in the radial and ulnar arteries and are less frequently observed in the periphery. As such, there are a limited number of reports describing aneurysms in the digits, and those involving the distal regions are even rarer [1,2]. Traumatic aneurysms are histologically classified into two distinct types; true aneurysms, in which the three-layered structure of the arterial wall is maintained, and pseudoaneurysms, which lack internal elastic plates and smooth muscle [3]. Additionally, pyogenic granuloma (PG), vascular lesions that also result from trauma, are also common. PGs are painful masses characterized by abnormal capillary proliferation that are easily hemorrhagic, and in adults, they generally occur on trauma-prone fingers. Both types of lesions seldom resolve spontaneously, and surgical treatment is often the treatment of choice [4]. As such, we performed surgical treatment of a vascular lesion on the lateral nail fold of a healthy adult. As the lesion had features of both traumatic pseudoaneurysm and PG on both physical and microscopic examination, we considered it to be an unusual case.

## Case report

A 28-year-old man with no pre-existing medical conditions was referred to our hospital for treatment of an erythematous nodular lesion on the lateral nail fold of the right middle finger. He had received blunt trauma to his right middle finger two months prior to the visit and had been suffering from a painful and easily hemorrhagic erythematous nodule on the lateral nail fold of the right middle finger since then. He was previously diagnosed with PG caused by an ingrown nail and was treated with oral and topical antibacterial agents. However, this did not result in an improvement in the condition and he was referred to our clinic because of a gradual increase in size.

**Figure 1.**
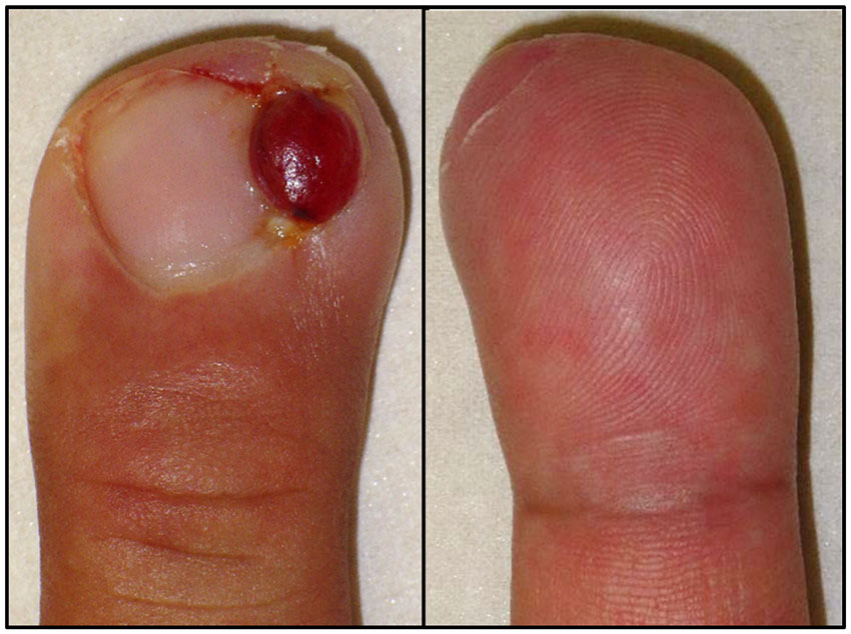
Appearance of the right middle finger at the time of initial examination. A smooth erythematous nodule 7 mm in size was observed on the ulnar lateral nail fold. The nail is shifted to the radial side. The subcutaneous area under the nodule was indurated, swollen, and tender. (left) Dorsal side of the affected finger. (right) Palmar side of the affected finger.

**Figure 2.**
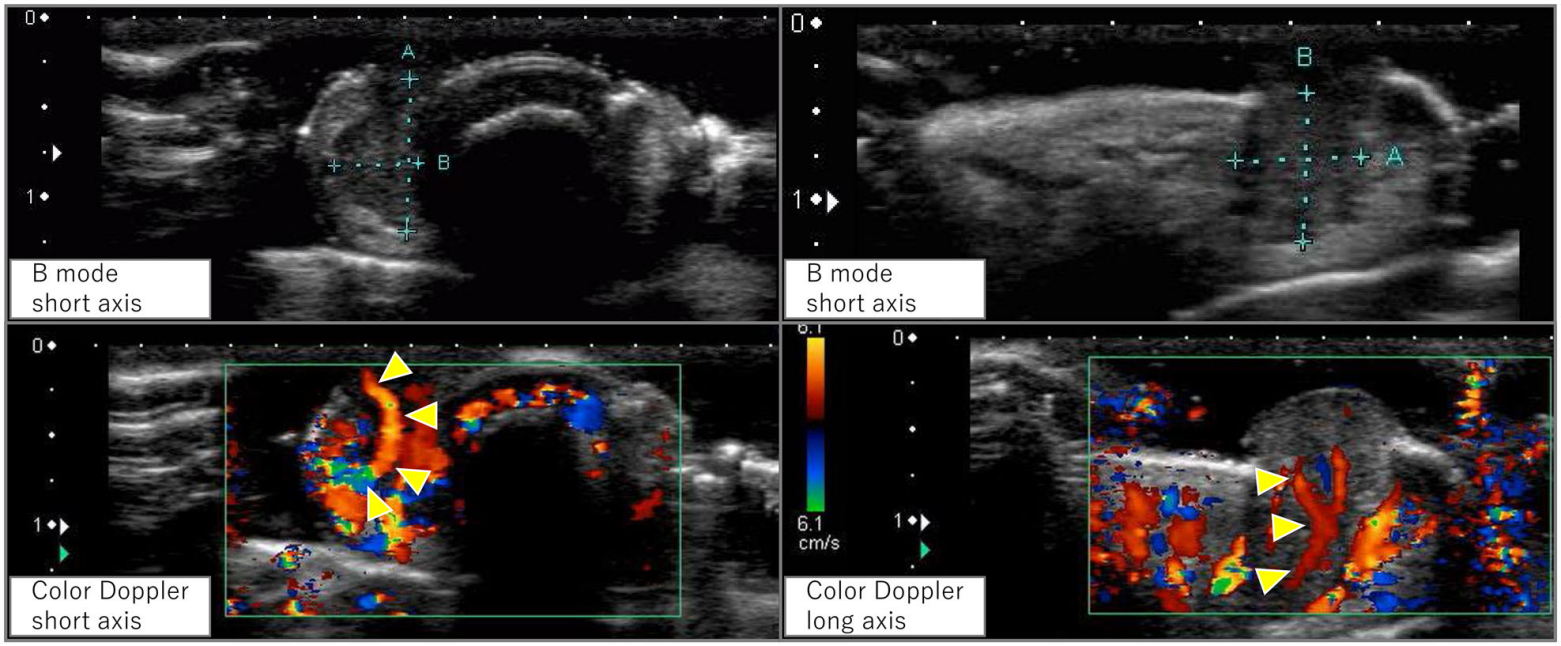
Ultrasound findings. B-mode images revealed a well-defined hypoechoic region seen protruding from the subcutis to the outside of the body. Color doppler images showed pulsatile vascular inflow from deep within the lesion (arrowhead).

**Figure 3.**
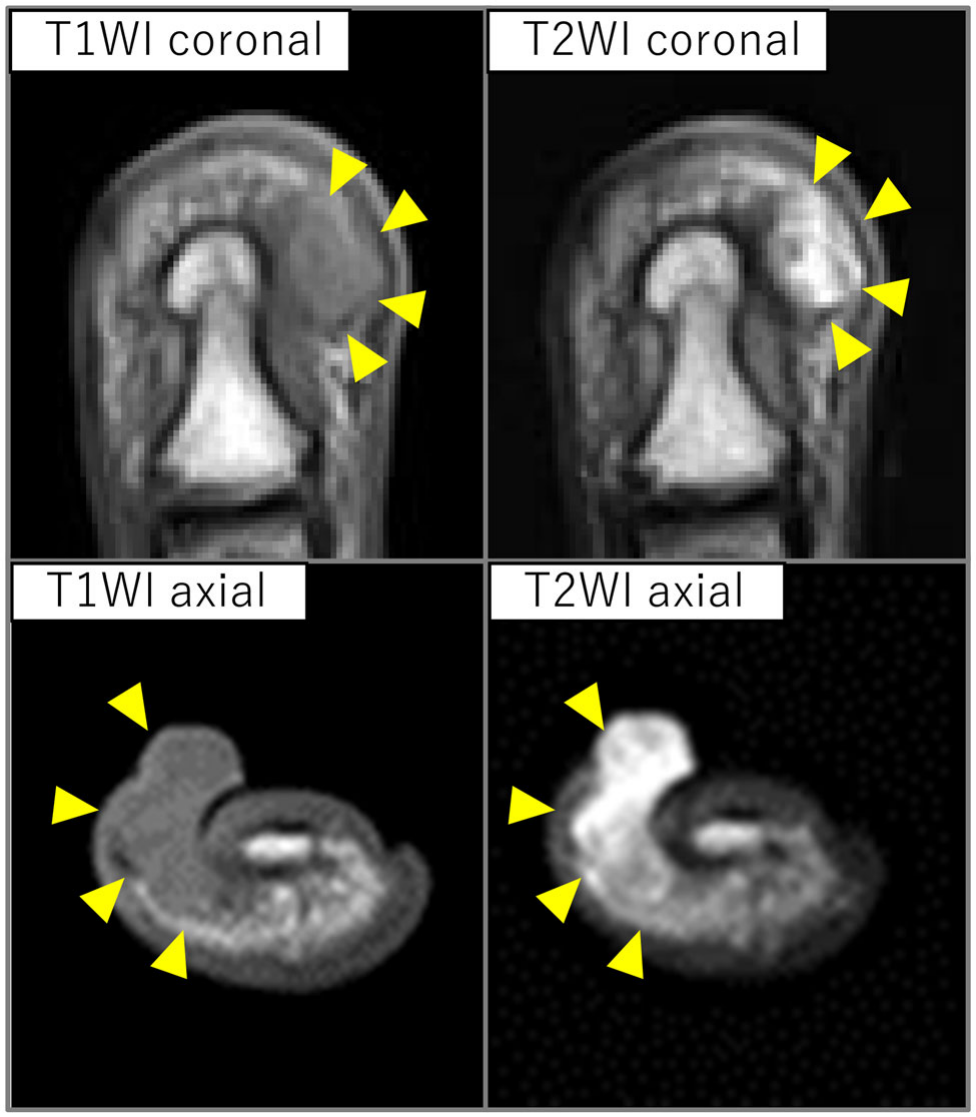
Magnetic resonance imaging findings. An iso-intensity in T1 and high-intensity in T2 weighted images lesion was observed protruding subcutaneously to the body surface on the ulnar aspect of the distal phalanx (arrowhead).

On initial examination, a soft, smooth, 7-mm-sized erythematous nodule was found on the ulnar lateral nail fold of his right middle finger. The subcutaneous area of the nodule was tender around the lesion with induration, with his nail shifted to the radial side (Figure 1). Ultrasonography revealed a borderline clear hypoechoic area extending continuously from the nodule to the subcutis of the fingertip (Figure 2). Furthermore, magnetic resonance imaging (MRI) showed a weighted images lesion of continuous iso-intensity in T1 and high-intensity in T2 on the ulnar aspect of the distal phalanx from subcutaneous through to outside the body (Figure 3). Based on these findings, we diagnosed him with a traumatic aneurysm and performed resection of the lesion under brachial plexus block.

**Figure 4.**
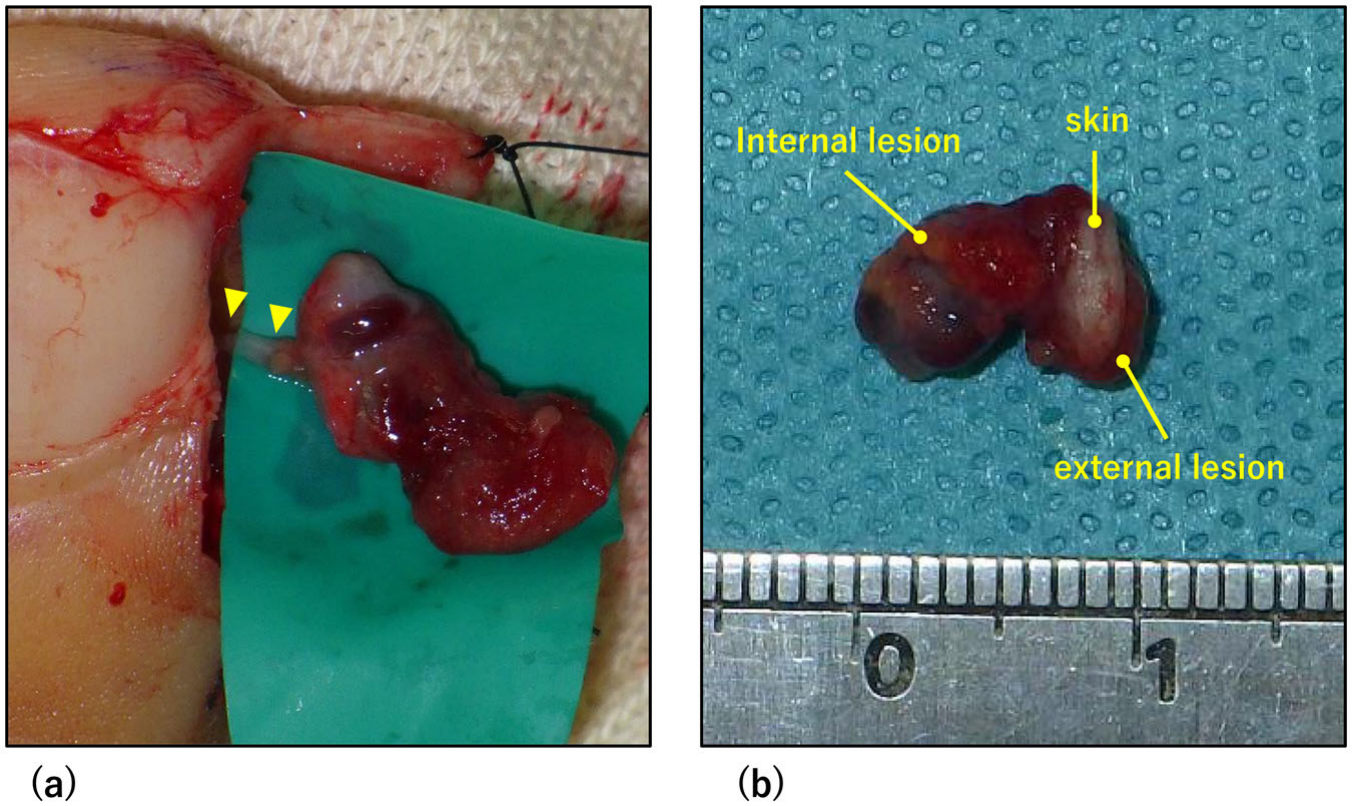
Intraoperative findings. (a) Intraoperative Photograph. A terminal branch of the digital artery flowed into the deep part of the lesion (arrowhead). (b) Photograph of the resected lesion. The lesion was hourglass-shaped, separated by the skin.

**Figure 5.**
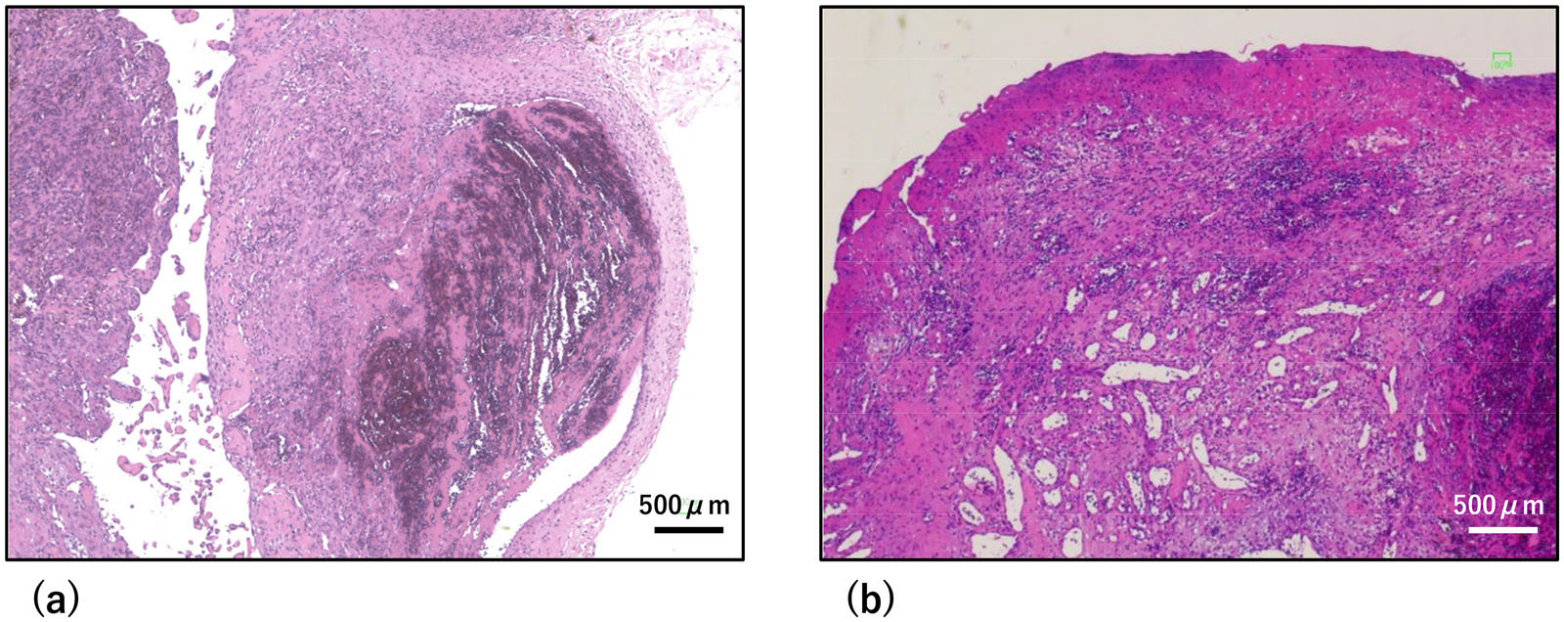
Histopathological findings. Hematoxylin-eosin stained images at 40x magnification. Scale bar = 500 µm. (a) Pathological findings for the lesion. The three-layered structure of the arterial wall was disrupted, consistent with the finding of a pseudoaneurysm. (b) Pathological findings of one region of the lesion. Capillary dilation and edematous stromal hyperplasia were observed, which were characteristic of pyogenic granuloma.

**Figure 6.**
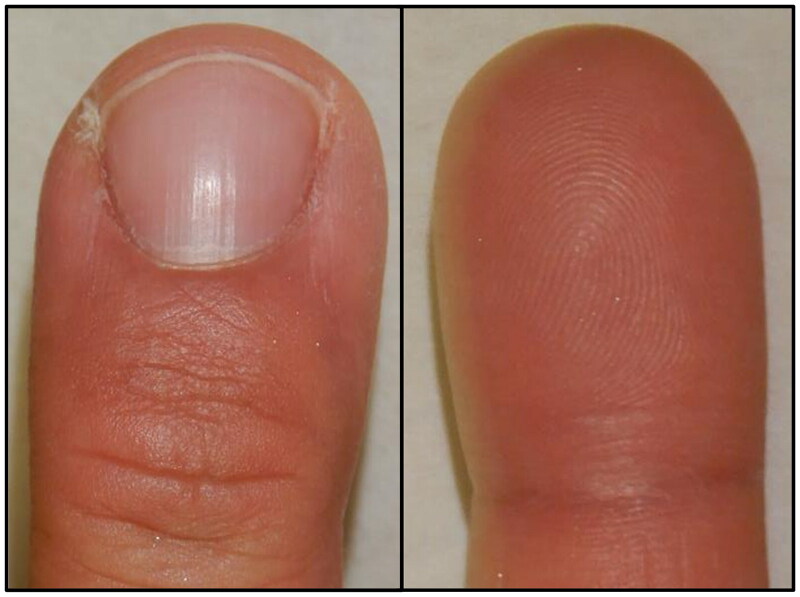
Appearance 6 months after surgery. No recurrence of lesions was noted, and finger morphology and nail shift improved.

The surgical approach involved a slight excision of the skin around the lesion. The lesion was hourglass-shaped, divided into internal and external parts by the skin, and was found to be in continuity with the terminal branches of the finger arteries in the deeper region of the lesion (Figure 4a, b). This inflow vessel was ligated, and the lesion was resected en bloc. Histopathologic examination revealed that the lesion had resulted in loss of the three-layer structure of the arterial wall and as such was diagnosed as a pseudoaneurysm (Figure 5a). Furthermore, some regions of the lesion also showed dilated capillaries and edematous stromal growth with features of PG (Figure 5b). His pain resolved quickly postoperatively, with no recurrence at 6 months, and his finger morphology and nail shift improved (Figure 6).

## Discussion

Traumatic aneurysms are broadly classified into true aneurysms and pseudoaneurysms based on their pathological properties. A true aneurysm occurs when the tunica media is damaged by contusion and the arterial wall is weakened and distended. The causes of true aneurysms include blunt trauma, chronic irritation, and vascular weakening due to an underlying health condition [5], and it is characterized histopathologically by the preservation of the three-layered structure of the arterial wall. Conversely, pseudoaneurysms are caused by damage to whole layers of the arterial wall, resulting in the formation of a hematoma around the vessel and subsequent formation of a capsule through coagulation and organization of the spilled blood [5]. They are caused by hematoma or infection following perforating vascular injury and are characterized histopathologically by a disruption of the three-layered structure of the arterial wall, as well as a deficiency of internal elastic plate and smooth muscle [1,3]. The diagnosis of a traumatic aneurysm is made through imaging studies, in addition to a history of underlying health conditions, trauma, and occupational history. Ultrasonography is a simple and useful method of imaging examination [1,6]. Specifically, computed tomography and MRI are used to evaluate localization and to differentiate the lesion from neoplastic lesions, while angiography and magnetic resonance angiography are used to evaluate the relationship with surrounding vessels if reconstruction is being considered [2,7]. There is currently no consensus on treatment, but surgery is generally the first choice [5], with early intervention desirable as lesions can expand over time. If the lesion develops centrally, reconstruction of the artery is performed as needed [2].

To date, there are very few reports describing aneurysms in the digits, and those involving the distal region are even rarer [1,2]. This may be due to the fact that although hand trauma itself is common, partial damage to the arterial wall is unlikely to occur due to the small vessel diameter [8]. Based on ultrasound and surgical findings, our case was thought to have arisen from a terminal branch of the digital artery, which may have been influenced by the specific environment at the edge of the nail.

In contrast, PG is one of the most common vascular abnormalities that occurs following trauma to the hands and fingers [4,9]. They are often observed as painful, easily hemorrhagic mass lesions with a propensity to increase in size, and are treated with topical therapy, cryotherapy, CO2 laser, and surgery [9]. Furthermore, the use of surgical treatment and subsequent histological studies for differential diagnosis is based on previous reports of malignancies that have a similar appearance to PG, such as amelanotic malignant melanoma and Kaposi’s sarcoma [10,11]. Despite being diagnosed by a previous physician as having treatment-resistant periungual PG, the physical and imaging findings for the patient described in this study were not consistent with pure PG. Several findings were indicative of a traumatic aneurysm while also presenting features of PG. Consequently, the histopathology showed a mixture of features of both traumatic pseudoaneurysm and PG, which were consistent with the physical, imaging and intraoperative findings. While it remains unclear how this lesion developed and expanded, but standard examination and treatment resulted in a positive outcome.

## Conclusions

We report the case of an unusual lesion demonstrating features of both a traumatic pseudoaneurysm and PG, originating from a terminal branch of a finger artery in the lateral nail fold of a 28-year-old man. When atypical PG is seen, ultrasound is simple and reliable to use in an outpatient setting.
